# SMOC1 colocalizes with Alzheimer’s disease neuropathology and delays Aβ aggregation

**DOI:** 10.1007/s00401-024-02819-6

**Published:** 2024-11-25

**Authors:** Kaleah Balcomb, Caitlin Johnston, Tomas Kavanagh, Dominique Leitner, Julie Schneider, Glenda Halliday, Thomas Wisniewski, Margaret Sunde, Eleanor Drummond

**Affiliations:** 1 Brain and Mind Centre and School of Medical Sciences, University of Sydney, Camperdown, NSW 2050 Australia; 2 School of Medical Sciences, University of Sydney, Camperdown, NSW 2050 Australia; 3 Center for Cognitive Neurology, Department of Neurology, Grossman School of Medicine, New York University, New York, NY 10016 USA; 4 Department of Neurology, New York University Grossman School of Medicine, New York, NY 10016 USA; 5 Rush Alzheimer’s Disease Center, Rush University Medical Center, 1750 W Harrison Street, Suite 1000, Chicago, IL 60612 USA; 6 Department of Neurological Sciences, Rush University Medical Center, Chicago, IL USA; 7 Department of Pathology, Rush University Medical Center, Chicago, IL USA; 8 Department of Pathology, New York University Grossman School of Medicine, New York, NY 10016 USA; 9 Department of Psychiatry, New York University Grossman School of Medicine, New York, NY 10016 USA

**Keywords:** SMOC1, Alzheimer’s disease, Beta amyloid, Tau, Tangles, Plaques, Preclinical, Mild cognitive impairment, Immunohistochemistry, Thioflavin T, Electron microscopy

## Abstract

SMOC1 has emerged as one of the most significant and consistent new biomarkers of early Alzheimer’s disease (AD). Recent studies show that SMOC1 is one of the earliest changing proteins in AD, with levels in the cerebrospinal fluid increasing many years before symptom onset. Despite this clear association with disease, little is known about the role of SMOC1 in AD or its function in the brain. Therefore, the aim of this study was to examine the distribution of SMOC1 in human AD brain tissue and to determine if SMOC1 influenced amyloid beta (Aβ) aggregation. The distribution of SMOC1 in human brain tissue was assessed in 3 brain regions (temporal cortex, hippocampus, and frontal cortex) using immunohistochemistry in a cohort of 73 cases encompassing advanced AD, mild cognitive impairment (MCI), preclinical AD, and cognitively normal controls. The Aβ- and phosphorylated tau-interaction with SMOC1 was assessed in control, MCI, and advanced AD human brain tissue using co-immunoprecipitation, and the influence of SMOC1 on Aβ aggregation kinetics was assessed using Thioflavin-T assays and electron microscopy. SMOC1 strongly colocalized with a subpopulation of amyloid plaques in AD (43.8 ± 2.4%), MCI (32.8 ± 5.4%), and preclinical AD (28.3 ± 6.4%). SMOC1 levels in the brain strongly correlated with plaque load, irrespective of disease stage. SMOC1 also colocalized with a subpopulation of phosphorylated tau aggregates in AD (9.6 ± 2.6%). Co-immunoprecipitation studies showed that SMOC1 strongly interacted with Aβ in human MCI and AD brain tissue and with phosphorylated tau in human AD brain tissue. Thioflavin-T aggregation assays showed that SMOC1 significantly delayed Aβ aggregation in a dose-dependent manner, and electron microscopy confirmed that the Aβ fibrils generated in the presence of SMOC1 had an altered morphology. Overall, our results emphasize the importance of SMOC1 in the onset and progression of AD and suggest that SMOC1 may influence pathology development in AD.

**Supplementary Information:**

The online version contains supplementary material available at 10.1007/s00401-024-02819-6.

## Introduction

SPARC-related modular calcium-binding protein 1 (SMOC1) has been shown to be one of the most dysregulated proteins in Alzheimer’s disease (AD) brain tissue, cerebrospinal fluid (CSF), and plasma in 23 proteomic analyses to date [8, 9, 21, 27, 28, 36, 45, 50, 53, 65, 66, 87, 88, 96, 98, 99, 102, 104, 115, 117, 119, 120, 127]. SMOC1 is significantly increased in human AD brain tissue, starting at the earliest preclinical stages and progressively increasing throughout disease [8, 21, 53, 65–67]. SMOC1 is significantly increased in the CSF of AD patients when compared to cognitively normal controls [27, 30, 36, 45, 48, 50, 53, 96, 109, 115, 117, 127] or other neurodegenerative diseases [29, 45, 77, 98, 107, 112, 120]. SMOC1 levels are significantly increased in the CSF 29 years before symptom onset in autosomal dominant AD [63, 108], making it one of the earliest altered proteins in AD [48]. SMOC1 is also one of the most enriched proteins in amyloid plaques in multiple subtypes of AD, including advanced AD, early onset AD, Down syndrome with AD, and preclinical AD [33, 122]. SMOC1 enrichment is observed in both sporadic and autosomal dominant AD in the brain [109], CSF [36, 63, 108, 109], and plasma [108]. SMOC1 is also significantly enriched in cerebral amyloid angiopathy (CAA) in comparison to blood vessels without CAA, suggesting that the SMOC1 association with amyloid beta (Aβ) is not limited to plaques [76, 120]. Proteomic studies have established that SMOC1 levels in the human brain and CSF strongly correlate with Aβ levels [9, 36, 48, 50, 96, 98, 102, 115, 117, 127] and SMOC1 has been identified as a strong AD-specific biomarker candidate either in isolation [48, 117, 127] or within a protein panel [48, 50, 87, 109]. SMOC1 is also significantly increased in blood serum [42] and plasma [49, 109] in AD, suggesting that it could be attractive blood biomarker for AD. When brain tissue, CSF, and blood plasma are combined, SMOC1 is the highest ranked biomarker of AD [115].

Despite the consistently reported link between SMOC1 and AD, little is known about the role of SMOC1 in the brain or in AD. SMOC1 is a secretory calcium-binding protein present throughout the brain, blood vessels, and pancreas [24, 44, 113]. SMOC1 is predominantly expressed by oligodendrocyte precursor cells (OPCs), with expression also observed in inhibitory vasoactive intestinal peptide interneurons, and oligodendrocytes at comparatively lower levels [10, 19, 60, 85, 105, 125]. SMOC1 contains two EF-hand calcium-binding sites [72, 113] which facilitate calcium-dependent binding to several proteins [18, 92], many of which are implicated in AD pathogenesis [59, 74, 89, 121]. SMOC1 plays a critical role in ocular, limb, and reproductive tract development [1, 95, 97], with genetic mutations causing Waardenburg Anophthalmia Syndrome [1, 80, 101]. SMOC1 has additionally been implicated in Chronic Kidney Disease [61], coronary artery disease [62], type II diabetes [31, 90], and various cancers [4–6, 116, 123].

Given the limited prior analysis of the distribution and role of SMOC1 in AD, the aims of this study were to perform a detailed neuropathological study of SMOC1 changes in human brain tissue throughout the spectrum of AD, to determine if SMOC1 interacted with Aβ or phosphorylated tau in human AD brain tissue, and to determine if SMOC1 influenced aggregation of Aβ.

## Methods

### Human tissue samples

Human brain tissue was acquired from Rush University (USA), the New York University Alzheimer’s Disease Center (USA), and the Sydney Brain Bank (Australia), which all provide human brain tissue from ethically approved longitudinally assessed regional brain donor programs on neurodegenerative diseases. Samples were obtained from multiple brain banks based on convenience and availability. Brain tissue was acquired under protocols with Institutional Review Board (IRB) approval at NYU Grossman School of Medicine, Rush University and the Southeastern Sydney and Illawarra Local Health District and the Universities of New South Wales and Sydney, Australia. In all cases, written informed consent for research was obtained from the patient or legal guardian, and the material used had appropriate ethical approval for use in this project. All patients’ data and samples were coded and handled according to NIH and NHMRC guidelines to protect patients’ identities.

Inferior temporal cortex tissue was obtained from Rush University, which was from cases part of the Religious Orders Study (ROS) and Memory and Aging Project (MAP) cohorts [12]. Clinical assessments and neuropathology were performed at Rush University [15, 79, 106]. Cases were initially stratified by the clinical cognitive final consensus diagnosis that was generated by a neurologist with expertise in dementia. The following neuropathological inclusion criteria were then used to refine case selection in each group: neuropathological ABC score of A0-1/B0-2/C0-1 for control, A2-3/B1-2/C2-3 for MCI, and A3/B3/C3 for AD. Control cases were further stratified into low-pathology controls and preclinical AD cases by staining for Aβ and identifying low or moderate Aβ plaque load, respectively. Cases were prioritized to exclude those with high TDP-43 and Lewy body pathology. Tissue included in this study from this cohort included 8 μm formalin-fixed paraffin-embedded (FFPE) sections of the inferior temporal cortex from control (*n* = 12), preclinical AD (*n* = 10), MCI (*n* = 12), and AD cases (*n* = 12).

Hippocampal tissue used in this study was obtained from New York University Alzheimer’s Disease Center (USA). This tissue included 8 μm FFPE sections containing the hippocampus from *n* = 7 AD and *n* = 4 cognitively unimpaired age-matched controls. Superior frontal cortex tissue was obtained from the Sydney Brain Bank (Australia). This included 8 μm FFPE sections from *n* = 10 AD and *n* = 7 cognitively unimpaired age-matched control cases and fresh-frozen tissue from *n* = 7 AD, *n* = 5 MCI, and *n* = 6 cognitively unimpaired age-matched controls. All groups were balanced for sex and postmortem interval where possible. Case-specific details are summarized in Table 1.

**Table 1:** Case information

Case ID	Brain region	Age	Sex	PMI	ABC score	Western blot characterization	SMOC1 IP	SMOC1/Aβ (4G8) IHC	SMOC1/pAβ IHC	SMOC1/pTau (AT8) IHC	Cell Type IHC
Control 1	SFC	93	F	21	A1 B0 C0	x		x			
Control 2	SFC	79	M	8	A0 B1 C0	x	x	x			
Control 3	SFC	89	F	23	A1 B1 C0	x	x	x			
Control 4	SFC	84	M	36	A1 B0 C0	x					
Control 5	SFC	89	M	27	A0 B2 C0	x					
Control 6	SFC	97	F	25	A1 B2 C0	x	x				
MCI 1	SFC	84	F	6	A3 B2 C2	x					
MCI 2	SFC	88	F	26	A3 B2 C2	x	x				
MCI 3	SFC	89	M	33	A3 B2 C1	x	x				
MCI 4	SFC	84	F	34	A3 B2 C2	x	x				
MCI 5	SFC	95	F	17	A3 B2 C2	x					
AD 1	SFC	80	F	32	A3 B3 C3	x					
AD 2	SFC	86	M	9	A3 B3 C3	x	x				
AD 3	SFC	85	F	10	A3 B3 C3	x	x				
AD 4	SFC	75	F	14	A3 B3 C3	x		x			
AD 5	SFC	92	M	21	A3 B3 C3	x	x				
AD 6	SFC	70	M	23	A3 B3 C3	x		x			
AD 7	SFC	73	M	26	A3 B3 C3	x		x			
Control 7	SFC	68	M	11	A0 B0 C0			x			
Control 8	SFC	90	F	58	A1 B0 C0			x			
Control 9	SFC	84	M	9	A1 B1 C1			x			
Control 10	SFC	93	F	15	A2 B1 C1			x			
AD 8	SFC	66	M	9	A3 B3 C3			x			
AD 9	SFC	91	F	6	A3 B3 C2			x			
AD 10	SFC	64	F	18	A3 B3 C3			x			
AD 11	SFC	70	M	8	A3 B3 C2			x			
AD 12	SFC	74	M	35	A3 B3 C3			x			
AD 13	SFC	69	M	19	A3 B3 C3			x			
Control 11	ITC	82	M	7	A1 B1 C0			x			
Control 12	ITC	90	F	26	A0 B1 C0			x			
Control 13	ITC	87	M	4	A0 B1 C0			x			
Control 14	ITC	95	F	5	A1 B1 C0			x			
Control 15	ITC	76	F	6	A1 B0 C0			x			
Control 16	ITC	91	F	15	A0 B2 C0			x			
Control 17	ITC	80	M	17	A0 B1 C0			x			
Control 18	ITC	81	F	5	A1 B1 C1			x			
Control 19	ITC	93	F	3	A0 B2 C0			x			
Control 20	ITC	83	F	1	A1 B1 C0			x			
Control 21	ITC	91	M	7	A1 B2 C0			x			
Control 22	ITC	84	M	11	A1 B1 C0			x			
Preclinical 1	ITC	89	F	27	A2 B2 C2			x			
Preclinical 2	ITC	80	M	27	A2 B2 C2			x			
Preclinical 3	ITC	85	F	5	A2 B2 C2			x			x
Preclinical 4	ITC	83	M	6	A2 B1 C1			x			
Preclinical 5	ITC	85	F	6	A2 B2 C2			x			
Preclinical 6	ITC	78	M	10	A1 B1 C1			x			
Preclinical 7	ITC	85	F	6	A1 B2 C1			x			
Preclinical 8	ITC	94	F	12	A2 B1 C1			x			
Preclinical 9	ITC	86	M	18	A1 B2 C2			x			
Preclinical 10	ITC	70	M	21	A2 B0 C2			x			
MCI 6	ITC	84	F	6	A2 B2 C1			x			
MCI 7	ITC	94	F	12	A2 B1 C2			x			
MCI 8	ITC	95	F	ND	A2 B2 C2			x			
MCI 9	ITC	84	M	5	A3 B2 C2			x			
MCI 10	ITC	85	M	21	A2 B2 C2			x			
MCI 11	ITC	89	M	5	A2 B2 C2			x			
MCI 12	ITC	75	M	16	A2 B2 C2			x			
MCI 13	ITC	91	F	13	A2 B2 C3			x			
MCI 14	ITC	85	F	18	A2 B2 C2			x			
MCI 15	ITC	88	F	6	A2 B3 C3			x			
MCI 16	ITC	98	F	7	A2 B2 C2			x			
MCI 17	ITC	89	F	20	A1 B2 C2			x			
AD 14	ITC	83	F	2	A3 B3 C3			x			
AD 15	ITC	81	F	6	A3 B3 C3			x			
AD 16	ITC	84	F	17	A3 B3 C3			x			x
AD 17	ITC	90	F	8	A3 B3 C3			x			
AD 18	ITC	88	F	26	A3 B3 C3			x			
AD 19	ITC	93	M	6	A3 B3 C3			x			
AD 20	ITC	87	M	9	A3 B3 C3			x			
AD 21	ITC	95	F	8	A3 B3 C3			x			x
AD 22	ITC	96	F	14	A3 B3 C3			x			
AD 23	ITC	87	M	4	A3 B3 C3			x			x
AD 24	ITC	96	M	5	A3 B3 C3			x			x
AD 25	ITC	72	F	3	A3 B3 C3			x			
Control 23	H	77	M	ND	A0 B0 C0			x		x	
Control 24	H	59	M	ND	A1 B0 C0			x		x	
Control 25	H	71	F	ND	ND			x		x	
Control 26	H	81	F	ND	ND			x		x	
AD 26	H	89	F	ND	A3 B3 C3			x	x	x	
AD 27	H	79	F	ND	A3 B3 C3			x	x	x	
AD 28	H	73	M	ND	A3 B3 C3			x	x	x	
AD 29	H	72	F	ND	A3 B3 C3			x	x	x	
AD 30	H	85	F	ND	A3 B3 C3			x		x	
AD 31	H	92	F	ND	A3 B3 C3			x	x	x	
AD 32	H	84	F	ND	A3 B3 C3			x		x	

*IP* immunoprecipitation, *IHC* immunohistochemistry, *PMI* postmortem interval, *SFC* superior frontal cortex, *ITC* inferior temporal cortex, *H* hippocampus, *ND* not determined

### Immunohistochemistry for image analysis

FFPE tissue sections underwent fluorescent immunohistochemistry using the method described in [34]. Briefly, sections were deparaffinized and rehydrated through a series of xylene and ethanol washes. Antigen retrieval was achieved using 99% formic acid for 7 min followed by boiling in citrate buffer for 21 min (10 mM sodium citrate, 0.05% Tween-20, pH 6). Sections were blocked in 10% normal goat serum and incubated with anti-SMOC1 (Abcam, ab200219, 1:100) combined with either anti-Aβ (BioLegend, 4G8, 800701, 1:1000), anti-pyroglutamated Aβ (BioLegend, 822301, 1:250), or anti-pTau (ThermoFisher, AT8, MN1020, 1:500) in 4% normal goat serum overnight at 4 °C. AlexaFluor488-, AlexaFluor647-conjugated secondary antibodies (Jackson ImmunoResearch Laboratories, 1:500) and Hoechst 33342 (Sigma, B2261, 1:1000) were applied for 2 h at room temperature prior to coverslipping with Antifade ProLong Glass (Invitrogen, P36984). Whole slide images were acquired using an Olympus VS200 Slide Scanner at 10× (NA 0.4, SMOC1/4G8) or 20× (NA 0.8, SMOC1/AT8) magnification. For SMOC1/pyroglutamated Aβ, whole slides were captured across multiple images on a Leica Thunder Fluorescence Microscope at 10× magnification (NA 0.32) with 10% stitching. Representative 60× images (NA 1.4, 2× zoom) were captured on a Nikon C2 Confocal microscope. Empty channel 568 was captured to allow for autofluorescence subtraction.

### Immunohistochemistry for cell type investigation

FFPE tissue sections underwent deparaffinization and rehydration as above, prior to boiling in citrate buffer for 21 min (10 mM sodium citrate, 0.05% Tween-20, pH 6). Sections were blocked in 10% normal horse serum and incubated with anti-SMOC1 (Abcam, ab200219, 1:100) and anti-Aβ (BioLegend, 4G8, 800701, 1:1000), combined with either anti-PDGFRa (R&D Systems, AF-307, 1:250), anti-Olig2 (R&D Systems, AF2418, 1:500), anti-GFAP (Novus Biologicals, NOVNBP1-05198, 1:1500), anti-Iba1 (Abcam, ab5076, 1:500), or anti-NeuN (Merck, ABN91, 1:500) in 4% normal horse serum overnight at 4 °C. CF488- (Sigma, SAB4600036, 1:1000), AlexaFluor594- (Jackson, 703-585-155, 1:1500), AlexaFluor647- (Thermo, A32849, 1:1000), AlexaFluor750- (Abcam, ab175739, 1:500) conjugated secondary antibodies and Hoechst 33342 (Sigma, B2261, 1:1000) were applied for 2 h at room temperature prior to coverslipping with Antifade ProLong Glass (Invitrogen, P36984). Whole slide images were acquired using an Olympus VS200 Slide Scanner at 40× magnification (NA 0.95). Representative 60× images (NA 1.4) were captured on a Nikon C2 Confocal microscope.

### SMOC1/4G8 immunohistochemistry analysis

SMOC1 load in amyloid plaques was assessed in temporal cortex, frontal cortex, and hippocampal sections. Grey matter regions of each frontal cortex and temporal cortex section were manually annotated in QuPath (v0.4.4). For hippocampal sections, the hippocampus was defined as the combined area containing CA1-4 and subiculum. A pixel classifier was trained in QuPath to recognize amyloid plaques and was used to generate a mask of all plaques in grey matter or hippocampus for each section. Images were exported as .tif images with corresponding plaque masks. Plaque masks were applied to images in ImageJ2 (v2.14.0), and empty channel 568 subtracted from 488 to minimize autofluorescent signal. For each cohort, an SMOC1 threshold was determined based on the negative control, and thresholded SMOC1 signal measured within masked regions.

### SMOC1/AT8 immunohistochemistry analysis

SMOC1 load in AT8-immunoreactive pTau lesions was assessed in hippocampal sections. Region annotation, image export, and background subtraction were performed in QuPath as above for the hippocampus (CA1-4 and subiculum). AT8-positive signal was masked in ImageJ2, and SMOC1 signal within masked regions was thresholded and measured.

### SMOC1/pyroglutamated Aβ immunohistochemistry analysis

SMOC1 load in pyroglutamated Aβ (pAβ) lesions was assessed in hippocampal sections. Hippocampus regions (CA1-4 and subiculum) were manually annotated in ImageJ2. pAβ-positive signal was masked in ImageJ2, and SMOC1 signal within masked regions thresholded and measured.

### Tissue homogenization

Tissue homogenization was performed as per [35]. Briefly, fresh-frozen frontal cortex tissue was pulverized and dounce homogenized in 20% w/v ice-cold homogenization buffer (50 mM HEPES pH 7.0, 250 mM sucrose, 1 mM EDTA, protease, and phosphatase inhibitor cocktails). Total brain homogenates were aliquoted and stored at −80 °C.

### Co-immunoprecipitation

2 μg of SMOC1 antibody (Abcam, ab200219) or rabbit IgG control antibody (Invitrogen, 02-6102) was added to 300 μg of total brain homogenate and brought up to 350 μL with homogenization buffer. Samples were incubated for 24 h at 4 °C with rotation (25 rpm) to allow antibody binding. Samples were then incubated with Dynabeads (1.5 mg/sample) and incubated on a rotator for 24 h at 4 °C. Elution was conducted by adding 20 μL of 1× LDS Sample Buffer to beads and incubating for 15 min (70 °C, 1000 rpm). The co-IP product was transferred to a clean tube and stored at −20 °C until use.

### Western blot analysis

Relative protein content of co-IP products and brain homogenates were visualized by western blot. Co-IP products were blotted using equal volumes per well. Brain homogenate total protein concentration was determined using Pierce BCA and normalized with deionized water. Samples were denatured by boiling at 95 °C for 5 min with DTT and LDS Sample Buffer. Proteins were resolved on 4–12% or 12% NuPage Bis–Tris gels and transferred to PVDF membranes. Membranes were blocked in 5% skim milk (Aβ, Rb pTau, PHF-1) or 5% skim milk with 5% NGS (SMOC1) in TBST and then incubated with anti-Aβ (CST 8243, 1:1000 dilution), anti-SMOC1 (Abcam 200219, 1:1000) or anti-PHF-1 (Peter Davies [47], 1:1000) overnight at 4 °C. Secondary HRP-linked IgG (Cytiva NA931V, NA934V, 1:25,000) was applied for 2 h and ECL signal recorded on an iBright CL1500 (Invitrogen).

### Recombinant Aβ_42_ and SMOC1 protein preparation

Aβ_42_ was prepared as per [110]. Briefly, recombinant lyophilized Aβ_42_ (rPeptide, A-1163-1) was resuspended in cold 50 mM NaOH to a concentration of 1 mg/ml, sonicated for 5 min in an ice bath, and passed through a 0.22 μm filter (10,000×*g*, 1 min) to remove larger aggregates. Filtrate concentration was determined via Nanodrop (A280, *ε* = 1490 cm^−1^ M^−1^) prior to storage at −20 °C for no longer than 1 month. To remove any large aggregates formed during thawing, thawed Aβ_42_ aliquots underwent an additional round of sonication and filtration, and concentration was re-measured as above.

Lyophilized recombinant His-tagged SMOC1 (Abcam, ab276453) was resuspended in ice-cold PBS and centrifuged at 16,000×*g* for 10 min at 4 °C to remove large aggregates. The supernatant was collected and dialyzed overnight against fresh PBS to eliminate contaminants. Protein concentration was determined via Nanodrop (A280, *ε* = 39,765 cm^−1^ M^−1^) prior to storage at −20 °C.

### Aβ_42_ Thioflavin-T assays

Thioflavin-T (ThT) assays were set up by initially adding neutralization buffer (100 mM phosphate buffer, pH 6.5) to each well of a 96-well half-area non-binding microplate (Corning 3881). SMOC1 or BSA at 0.125–1.25 μM was added prior to addition of Aβ_42_ at a final concentration of 2.5 μM. Control samples with SMOC1 or BSA alone at 1.25 μM were included to assess baseline ThT fluorescence. Plates were immediately transferred to a POLARstar Omega microplate reader (BMG Labtech) at 37 °C. ThT fluorescence was measured at Ex/Em 440/480 nm for 50 h under quiescent conditions. Lag times were calculated as per [25, 91]. Post-assay, samples were processed for SDS-PAGE and TEM analysis.

### Preparation of mature Aβ_42_ fibrils

Aβ_42_ in 50 mM NaOH was neutralized (100 mM phosphate buffer, pH 6.5) to a final concentration of 20 μM and incubated for 32 h at 37 °C. Following incubation, fibrils were diluted to a final concentration of 10 μM in the presence or absence of SMOC1 or BSA at a molar ratio of 10:1. SMOC1 and BSA alone at 1 μM were included as additional controls. All samples were incubated for 2 h at RT prior to SDS-PAGE and TEM analysis.

#### SDS–PAGE

A portion of each ThT assay sample was reserved (‘Total’) with the remaining solution centrifuged at 4 °C (80,000×*g*, 20 min). The supernatant was collected (‘Soluble fraction’) and the pellet resuspended in 8 M urea (‘Pellet fraction’). Samples were denatured by boiling at 95 °C for 5 min with DTT and Novex Tricine SDS Sample Buffer (ThermoFisher, LC1676). Proteins were resolved on NuPAGE 10–20% Tricine Protein Gels, stained with Sypro-Ruby protein gel stain, and imaged using an iBright CL1500 (Invitrogen).

### Transmission electron microscopy (TEM)

5 μL of each sample was applied to a glow-discharged, standard Formvar on carbon film copper TEM grids (ProScitech, GSFC100CU) for 2 min. Excess liquid was removed with filter paper, and grids washed three times with water. Each grid was incubated with 10 μL of 10 nm Ni–NTA-Nanogold (Nanoprobes, 2084-3ML, 1:50) followed by 3× buffer washes (20 mM Tris, pH 7.6, 150 mM NaCl, 8 mM imidazole) as per the manufacturer’s instructions. Grids were stained with 2% uranyl acetate solution for 2 min and air-dried overnight. Replicate samples were imaged by TEM and showed consistent morphology. For analysis of fibril length, *n* ≥ 20 TEM images per sample were collected using an FEI Tecnai T12 at 120 kV and fibril lengths calculated using ImageJ.

### Statistical analysis

All results were analyzed in GraphPad Prism (v10.0.3). Each dataset was assessed for Gaussian distribution using a Shapiro–Wilk normality test. For immunohistochemistry analysis, normally distributed datasets were analyzed using Brown–Forsyth and Welch ANOVA tests with Dunnett’s T3 post-hoc, or Pearson correlation where appropriate. Non-normal datasets were analyzed using Kruskal–Wallis test with Dunn’s post hoc, nonparametric Spearman correlation or Mann–Whitney two-tailed *U* test where applicable. Statistics were not performed on pAβ/4G8 staining comparisons due to differences in image analysis methods. Thioflavin-T assay results were fitted with Boltzmann sigmoidal curves and analyzed using an ordinary two-way ANOVA with Tukey’s post hoc. Aβ_42_ fibril lengths were analyzed by a Kruskal–Wallis test with Dunn’s post hoc.

## Results

### SMOC1 colocalizes with a subset of Aβ plaques in early and advanced AD

Our first aim was to determine how early SMOC1 accumulates in the brain in AD. To determine this, we performed immunohistochemistry on temporal cortex FFPE sections from *n* = 12 AD, *n* = 12 MCI, *n* = 10 preclinical AD, and *n* = 12 age- and sex-matched control cases to determine when SMOC1 accumulates in AD, and if SMOC1 colocalizes with amyloid plaques in early stages of AD.

**Fig. 1:**
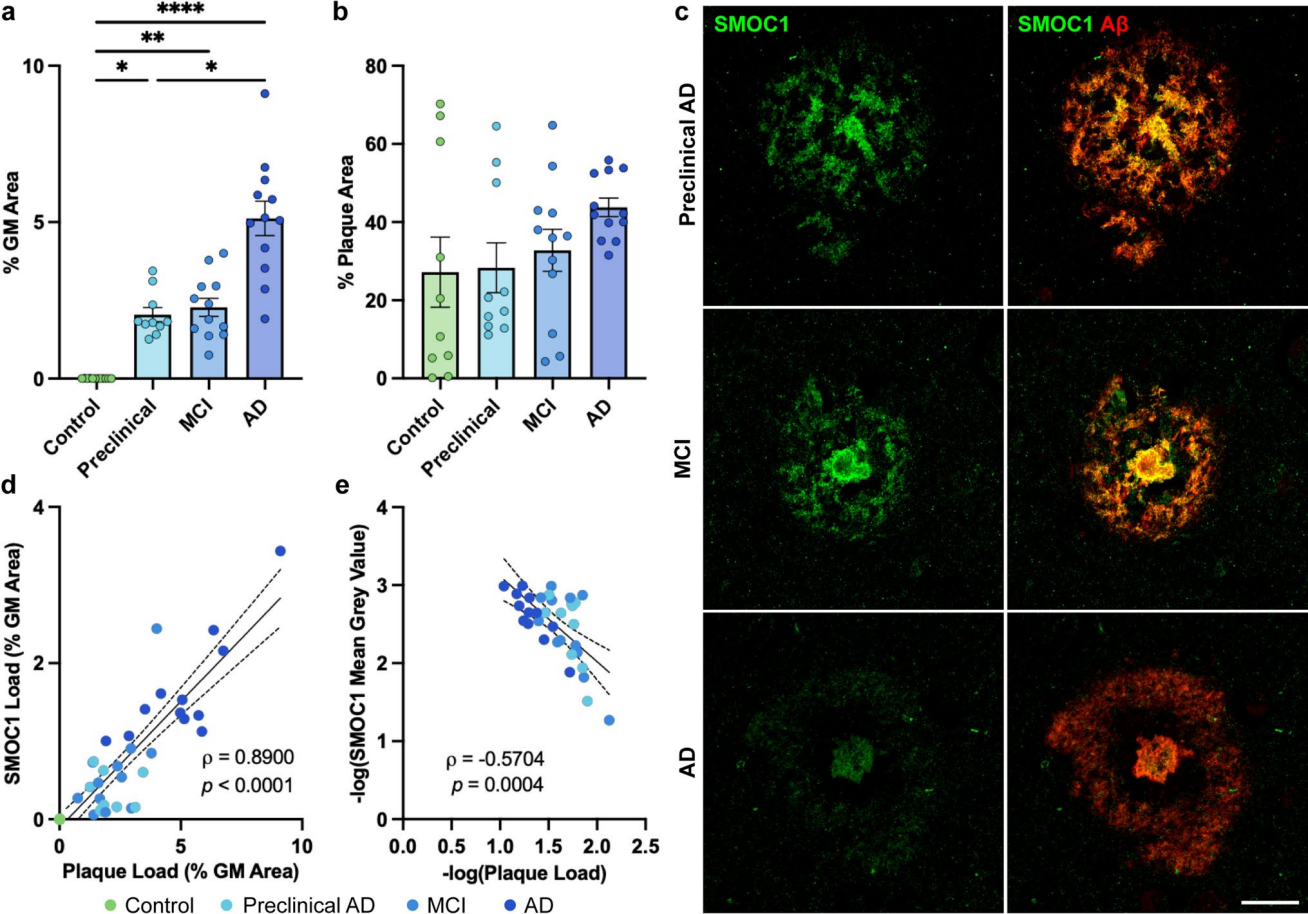
Characterization of SMOC1 colocalization in amyloid plaques in progressive stages of AD. **a** Amyloid plaque load increased with disease stage, with preclinical AD and MCI showing similar plaque abundance. Data show mean ± SEM of *n* = 10 preclinical AD, *n* = 12 control, MCI, AD; * *p* < 0.05, ** *p* < 0.01, **** *p* < 0.0001 determined by a Kruskal–Wallis test with Dunn’s post hoc analysis. **b** Percentage of amyloid plaques that colocalized with SMOC1 in each disease stage. **c** Representative images showing SMOC1 colocalization pattern of SMOC1 in amyloid plaques at each disease stage. **d** SMOC1 immunofluorescence in plaques strongly correlated with amyloid plaque load, independent of disease stage (nonparametric Spearman correlation). **e** The average intensity of SMOC1 immunofluorescence in plaques decreased as amyloid plaque load increased (Pearson correlation). Dotted lines represent 95% confidence intervals. GM, grey matter. Scale bar = 20 μm

To first determine the extent of pathology within our disease cohort, amyloid plaque load was assessed in temporal cortex grey matter. As expected, plaque load was significantly higher at all disease stages compared to control (*p* < 0.02). Preclinical AD and MCI showed similar levels of amyloid pathology (cortical loads of 2.0 ± 0.2% and 2.3 ± 0.3%, respectively, *p* > 0.999). Amyloid plaque load was significantly increased in AD cases (5.1 ± 0.5%) compared to control (*p* < 0.001) and preclinical AD (*p* = 0.038) but not MCI (*p* = 0.061), with increased variation between cases being observed as disease progressed (Fig. 1a).

A trained classifier was used to identify all amyloid plaques in each section and SMOC1 immunoreactivity within amyloid plaques was measured. We found no significant differences in SMOC1-positive plaque loads between AD stages (*p* > 0.263): 28.3 ± 6.4% of plaques in preclinical AD, 32.8 ± 5.4% of plaques in MCI, and 43.8 ± 2.4% of plaques in AD (Fig. 1b, c). Interestingly, SMOC1 also colocalized with a similar subpopulation (27.2 ± 9.0%) of the small number of amyloid plaques present in control cases (amyloid load: 0.003 ± 0.001%). The inter-patient variation in SMOC1 colocalization in amyloid plaques decreased with advancing disease stage, suggesting that SMOC1 colocalization in plaques may plateau as disease progresses.

Given the variation observed in amyloid plaque load in our cohort, particularly in AD, we next evaluated SMOC1 colocalization in amyloid plaques independent of disease stage. This analysis showed that total SMOC1 immunoreactivity in amyloid plaques strongly correlated with plaque load, irrespective of disease stage (Spearman’s *⍴* = 0.890, *p* < 0.0001) (Fig. 1d). The intensity of SMOC1 staining within amyloid plaques showed an inverse relationship to plaque load (Spearman’s *⍴* = −0.570, *p* = 0.0004) (Fig. 1e), indicating that SMOC1 expression in plaques is most concentrated when initial plaque pathology appears.

**Fig. 2:**
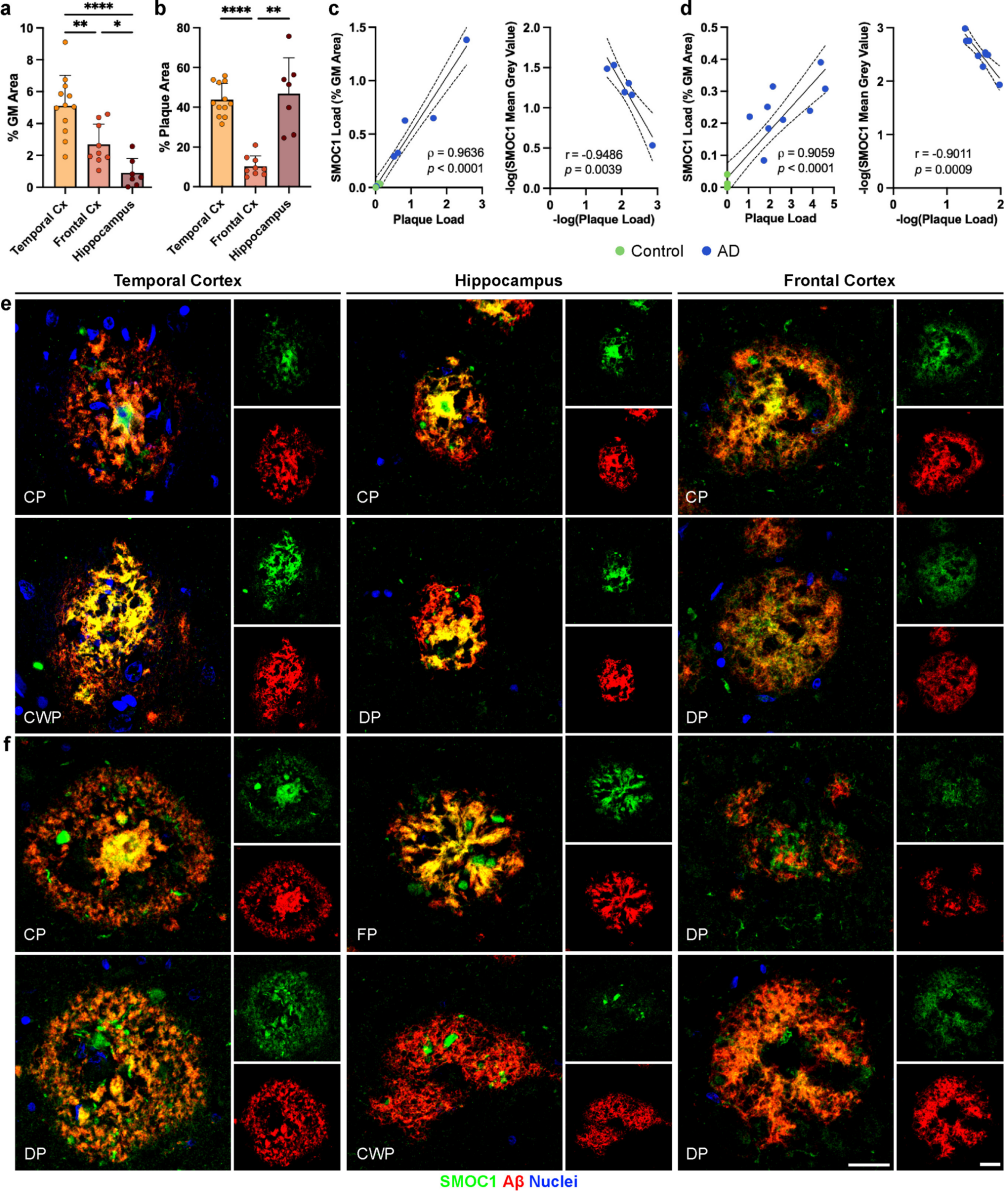
Characterization of SMOC1 colocalization in amyloid plaques in three brain regions. **a** Amyloid plaque load in advanced AD cases was decreased in the frontal cortex and hippocampus compared to the temporal cortex in our cohort. **b** SMOC1 colocalized with a subpopulation of amyloid plaques in all regions; however, SMOC1 colocalization in plaques was significantly lower in the frontal cortex compared to the temporal cortex and hippocampus. Data show mean ± SEM of *n* = 12 temporal cortex, *n* = 7 hippocampus, *n* = 9 frontal cortex; ** *p* < 0.01, *** *p* < 0.001, **** *p* < 0.0001 determined by a Kruskal–Wallis test with Dunn’s post-hoc analysis. SMOC1 immunofluorescence in plaques in both the hippocampus (**c**) and frontal cortex (**d**) strongly correlated with amyloid plaque load, showing a decrease in the average intensity of SMOC1 immunofluorescence as amyloid plaque load increased similar to that observed in the temporal cortex. Nonparametric Spearman correlation, Pearson correlation, dotted lines represent 95% confidence intervals. Representative immunofluorescence images showing the diversity of SMOC1 colocalization in plaques. In all regions, some plaques could be observed in which SMOC1 appeared to be coating fibrils (**e**), while other plaques showed SMOC1 presence in plaques distinct of amyloid immunoreactivity (**f**). Cx, cortex, GM, grey matter; CP, cored plaque; CWP, cotton wool plaque; DP, diffuse plaque; FP, fibrillar plaque. Scale bar = 20 μm

Having shown that SMOC1 colocalized with plaques in all stages of AD within the temporal cortex, we next aimed to determine if SMOC1 similarly colocalized with amyloid plaques in other brain regions, as this has not been previously explored. To do this, we performed an immunohistochemistry study using hippocampal (*n* = 7 AD, *n* = 4 control) and frontal cortex sections (*n* = 9 AD, *n* = 7 control) from a separate cohort of cases. Despite significantly different total amyloid plaque loads in the temporal cortex and hippocampus (*p* < 0.0001) (Fig. 2a), a similar degree of SMOC1 colocalization with amyloid plaques was observed (43.8 ± 2.4% and 46.9 ± 6.8%, respectively, *p* = 0.962) (Fig. 2b). In contrast, SMOC1 colocalized with a significantly smaller proportion of amyloid plaques in the frontal cortex (10.4 ± 1.7%) compared to the temporal cortex (*p* < 0.0001) or hippocampus (*p* = 0.0036). Similarly to the temporal cortex, SMOC1 colocalization and average intensity in amyloid plaques strongly correlated with plaque load in the hippocampus (Spearman’s *⍴* = 0.964, *p* < 0.0001; Pearson’s *r* = −0.949, *p* = 0.0039) (Fig. 2c) and frontal cortex (Spearman’s *⍴* = 0.892, *p* < 0.0001; Pearson’s *r* = −0.724, *p* = 0.0179) (Fig. 2d).

In all brain regions assessed, SMOC1 immunoreactive plaques showed a range of morphologies, including diffuse, cotton wool, lake-like, and cored, suggesting that these different morphologies did not define the subpopulation of plaques immunoreactive for SMOC1 (Fig. 2e, f). Plaques of the same morphology displayed a range of SMOC1 immunoreactivity, with SMOC1 colocalization variably evident in none, part, or all of plaques with the same morphology. Cored plaques positive for SMOC1 consistently contained SMOC1 in the core, but did not always show SMOC1 positivity in the corona of the plaque. The pattern of SMOC1 immunoreactivity within plaques was also diverse within individuals, with some plaques exhibiting highly specific colocalization between SMOC1 and Aβ (Fig. 2e), while other plaques showed SMOC1 immunoreactivity in the gaps between amyloid fibrils (Fig. 2f). Plaques exhibiting SMOC1 in fibril gaps were less prevalent in frontal cortex, possibly contributing to the lower proportion of SMOC1 colocalization in amyloid plaques observed in this region.

### SMOC1 colocalization with Aβ plaques is not driven by pyroglutamated Aβ

**Fig. 3:**
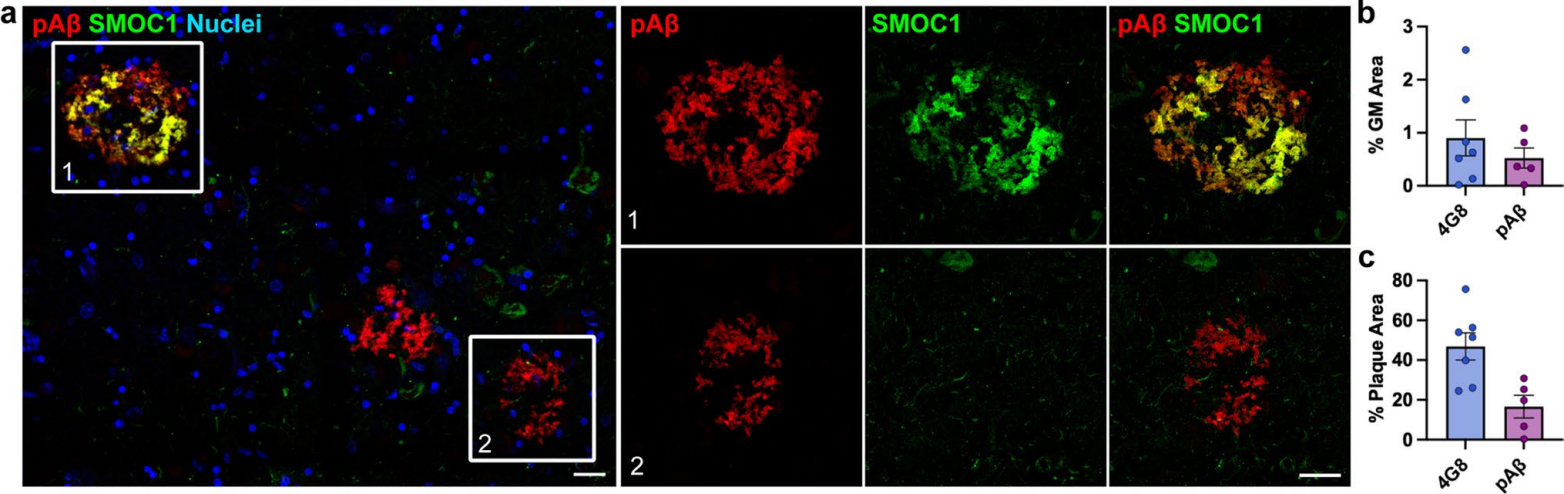
SMOC1 colocalization in amyloid plaques is not defined by presence of pyroglutamated amyloid β. **a** SMOC1 immunoreactivity was only observed in a subset of pyroglutamated Aβ plaques in the hippocampus. Boxed regions show examples of SMOC1-positive plaques that were pyroglutamated Aβ positive (1) and negative (2). **b** Quantification of plaque load immunoreactive for pyroglutamated Aβ in comparison to 4G8 in hippocampal sections. **c** SMOC1 colocalization in plaques immunoreactive for pyroglutamated Aβ and 4G8 in hippocampal sections. pAβ, pyroglutamated amyloid β; 4G8, anti-amyloid antibody reactive to residues 17–24. Scale bars = 20 μm

We were next interested to explore why only a subset of amyloid plaques showed SMOC1 immunoreactivity. As mentioned above, SMOC1 colocalization in amyloid plaques was not unique to plaques with a particular morphology. SMOC1 immunoreactivity in plaques did not correlate to plaque size (*r* = 0.2460, *p* = 0.1748) (Supplementary Fig. 1), indicating that SMOC1 specificity was not determined by the size of amyloid plaque. Based on our previous preliminary observations [33], we next hypothesized that SMOC1 specifically colocalized with plaques containing the pyroglutamated form of Aβ at Glu3. To determine if this was the case, double immunofluorescence for SMOC1 and pAβ was conducted on *n* = 5 AD hippocampal sections. Surprisingly, while some pAβ plaques were SMOC1 positive, many pAβ + plaques were devoid of SMOC1 staining (Fig. 3a). As expected, plaque load for pAβ was lower than 4G8 (Fig. 3b); however, SMOC1 colocalized with a considerably lower proportion of pAβ immunoreactive plaques (16.6 ± 5.7%) in comparison to 4G8 immunoreactive plaques (46.9 ± 6.8%) (Fig. 3c). It is, therefore, unlikely that pAβ is the determining factor for SMOC1 plaque colocalization.

### SMOC1 immunoreactivity is observed in blood vessels, spongiform, and small cells

**Fig. 4:**
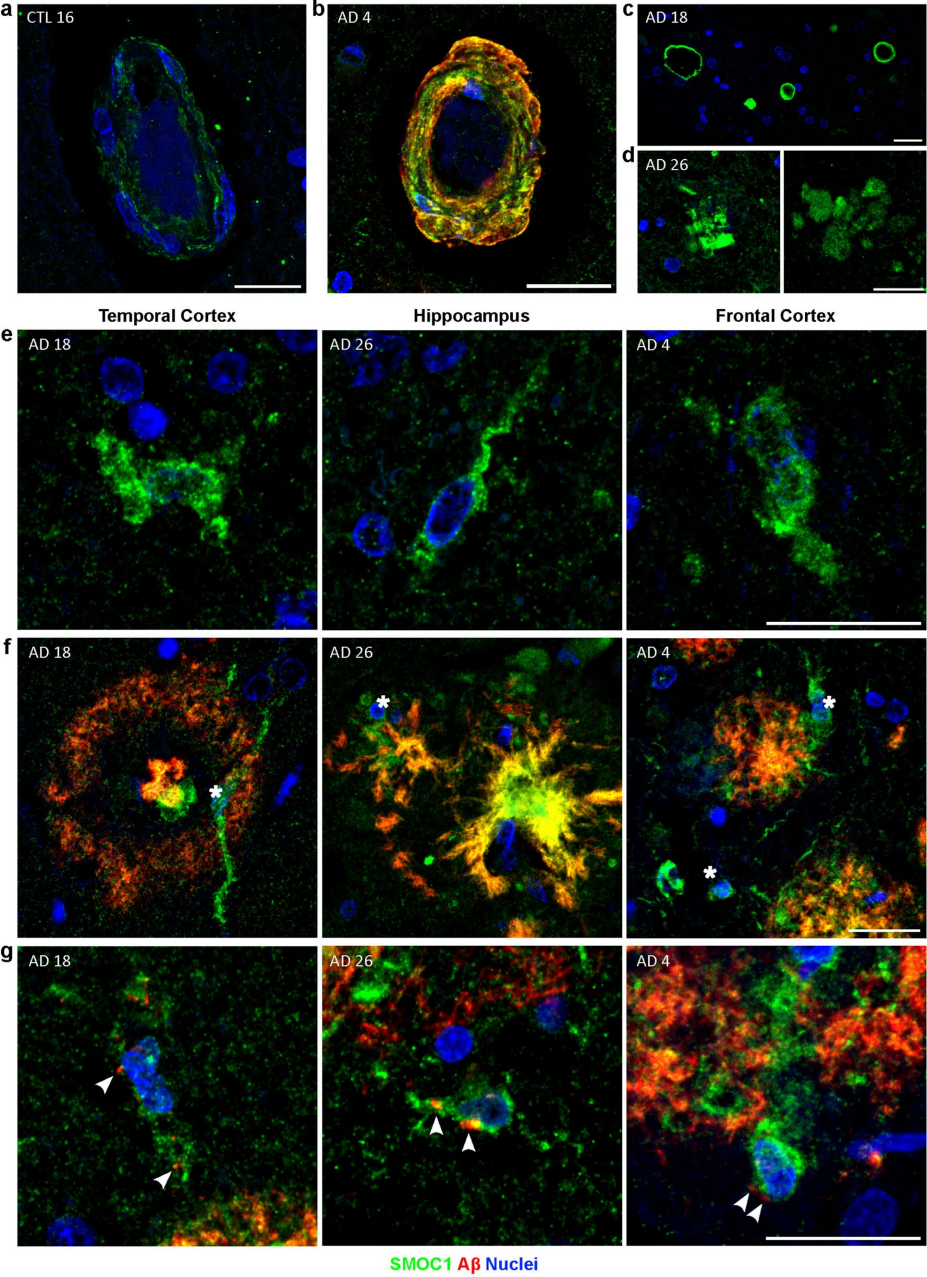
Diverse SMOC1 immunoreactivity in the human brain. **a** SMOC1 was observed at a low level in blood vessels in all regions and disease stages assessed, including controls. **b** SMOC1 colocalized with cerebral amyloid angiopathy. **c** SMOC1 clearly marked spongiform. **d** Example images showing SMOC1-positive formations in the advanced AD hippocampus with similar morphology to amyloid plaques, in the absence of Aβ immunoreactivity. Example images showing occasional SMOC1-positive cells in the white matter (**e**) and the grey matter (**f**). In the grey matter, these cells were often adjacent to amyloid plaques (stars). (**g**) Example images showing SMOC1 immunoreactive cells containing intracellular amyloid puncta (arrowheads). Scale bars = 20 μm

As detailed above, the most predominant SMOC1 immunoreactivity in all sections was observed in amyloid plaques. However, a diverse array of additional staining patterns was also observed with lower SMOC1 immunoreactivity. For example, a low level of diffuse SMOC1 staining was evident in white matter in all cases. SMOC1 staining was also observed in most blood vessels, localized to vessel walls throughout the parenchyma and leptomeninges (Fig. 4a). SMOC1 immunoreactivity in blood vessels was notably increased when CAA was present (Fig. 4b), consistent with our recent findings [76]. SMOC1 immunofluorescence was observed in all CAA phenotypes, independent of accompanying Aβ plaque pathology, notably including SMOC1 colocalization with CAA in a control case (Control 14) that had no Aβ plaque pathology. Co-localization of SMOC1 with Aβ was evident in leptomeningeal and cortical vessels ranging in size, including both capillaries and small arteries.

SMOC1 clearly stained spongiform that was variably present in our cohort (Fig. 4c). Interestingly, some localized accumulations of SMOC1 were also observed in the hippocampus that had a similar morphology to amyloid plaques, despite the lack of Aβ present (Fig. 4d). These were observable in all hippocampal AD sections in low numbers and were predominantly localized to CA1-subiculum. Small cells showing SMOC1 positivity in the soma were also observable within both the grey and white matter (Fig. 4e). These were evident at all stages of AD, but were most apparent in cases with low plaque load and were infrequently observed in control cases. Where amyloid pathology was present, cells were often observed adjacent to amyloid plaques (Fig. 4f). Occasionally, cellular processes could be observed extending into plaques, with cell bodies containing small Aβ puncta (Fig. 4g).

### SMOC1 is predominantly expressed by Olig2-negative oligodendrocyte precursor cells

**Fig. 5:**
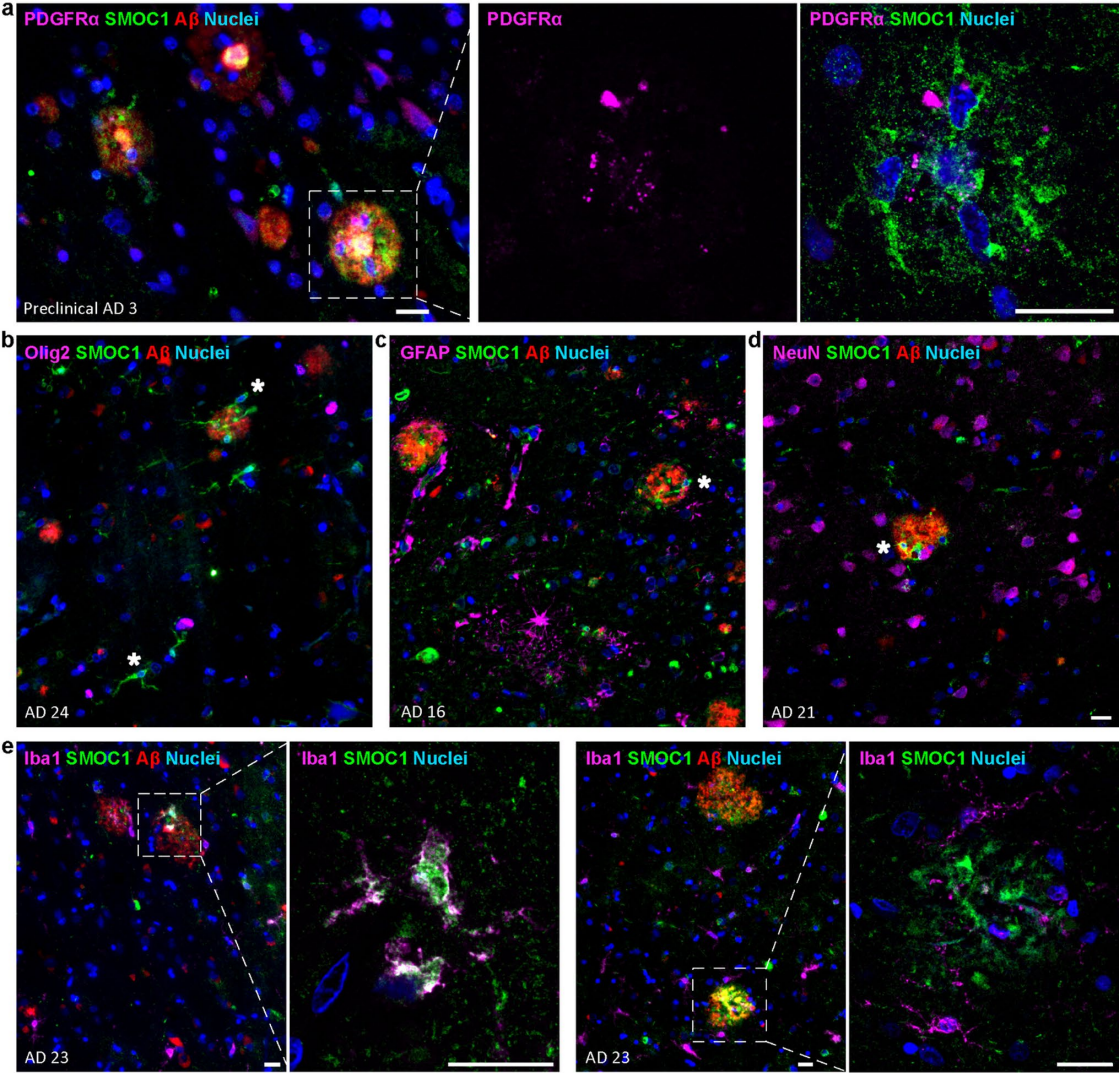
Characterization of SMOC1 immunoreactive cells. **a** SMOC1 immunoreactive cells were frequently positive for PDGFRα. High magnification image of a plaque (boxed) shows PDGFRα staining alone and with SMOC1-positive cells (arrows). SMOC1-positive cells (stars) were negative for Olig2 (**b**), GFAP (**c**), and NeuN (**d**). **e** Iba1-positive cells occasionally showed SMOC1 immunoreactivity. Cells positive for both Iba1 and SMOC1 around plaques (boxed) are shown in high magnification. Scale bars = 20 μm

Given the proximity of SMOC1-positive cells to amyloid plaques, we sought to identify which cell type was expressing SMOC1 in AD. Temporal cortex sections containing consistent SMOC1 cellular staining and abundant amyloid plaques were co-stained for SMOC1, Aβ, and markers for OPCs (PDGFRα), oligodendrocytes (Olig2), astrocytes (GFAP), microglia (Iba1), and neurons (NeuN). Co-localization of SMOC1 and PDGFRα was frequently observed (Fig. 5a) indicating that SMOC1 expression is highest in OPCs, as expected from RNAseq datasets [22, 38, 46, 60, 83–85, 105]. Interestingly, SMOC1-positive cells were negative for Olig2 (Fig. 5b), indicating that SMOC1 expression in AD may be specific to a subpopulation of OPCs negative for this marker [39, 40]. No colocalization was observed for astrocytes (Fig. 5c) or neurons (Fig. 5d). Iba1-positive microglia occasionally showed SMOC1 immunoreactivity (Fig. 5e); however, this was observed infrequently and to a lesser extent than OPCs.

### SMOC1 colocalizes with tau

**Fig. 6:**
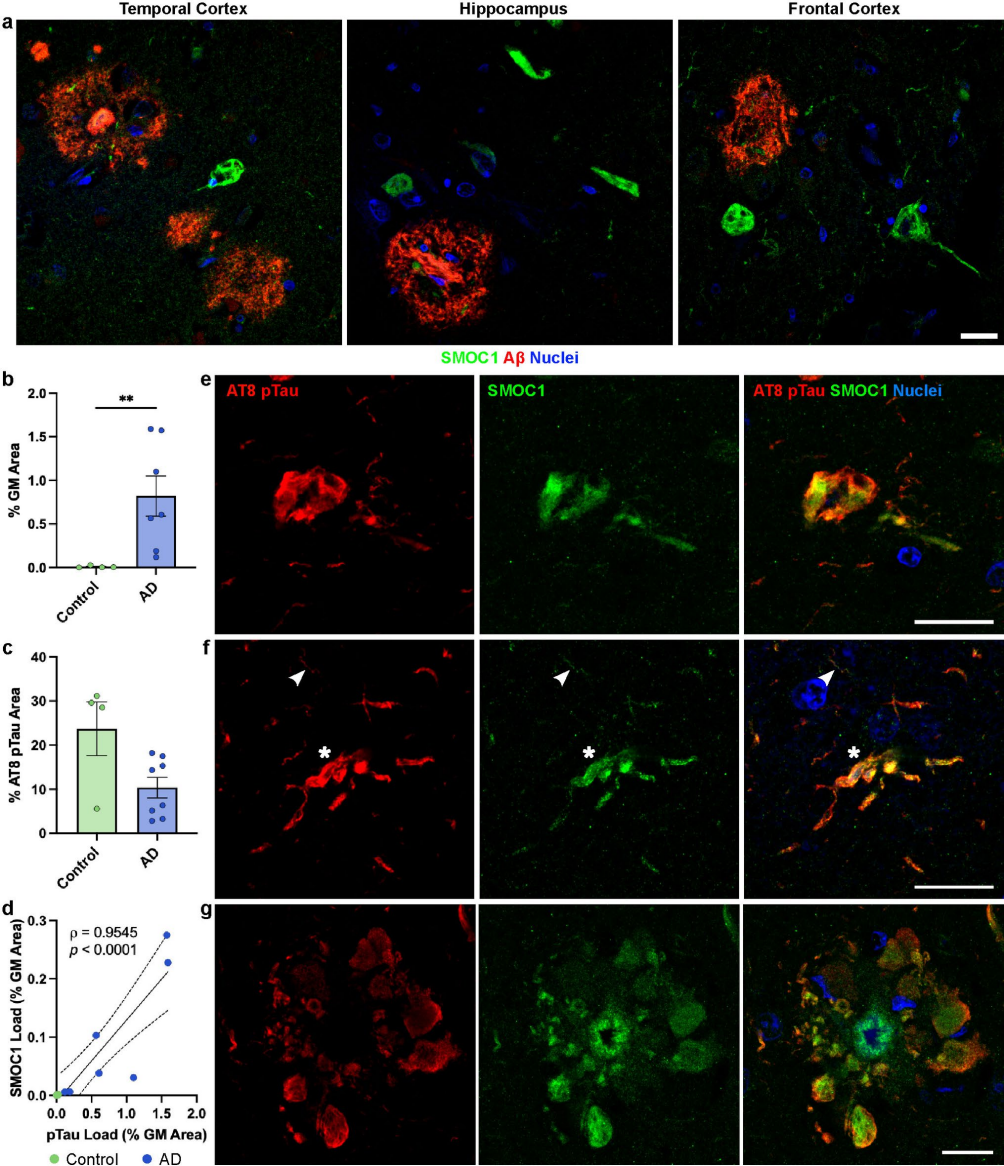
SMOC1 colocalization with pTau in Alzheimer’s disease. **a** SMOC1 immunofluorescence was observed in structures resembling neurofibrillary tangles in the temporal cortex, hippocampus and frontal cortex of AD cases. **b** Quantification of AT8-immunoreactive pTau load in hippocampal sections in our cohort. **c** SMOC1 colocalized with a subpopulation of AT8-immunoreactive pTau lesions in both control and advanced AD cases. Data show mean ± SEM of *n* = 4 control and *n* = 7 AD cases; ** *p* = 0.01 determined by Mann–Whitney *U* test. **d** SMOC1 colocalization with AT8-immunoreactive pTau strongly correlated with total pTau load, determined by nonparametric Spearman correlation. Representative images show SMOC1 colocalization with AT8-immunoreactive neurofibrillary tangles (**e**), dystrophic neurites (**f** stars), neuropil threads (**f** arrowheads), and neuritic plaques (**g**) in hippocampal sections. pTau, phosphorylated tau; GM, grey matter. Scale bars = 20 μm

SMOC1 also appeared to colocalize with a subpopulation of neurofibrillary tangles in all regions (Fig. 6a). This was unexpected given that SMOC1 has not been indicated as a tau interactor in previous proteomic studies [35, 54]. To confirm these observations and determine the prevalence of SMOC1 colocalization with phosphorylated tau, *n* = 4 control and *n* = 7 AD hippocampal FFPE sections were co-stained for SMOC1 and AT8 (pTau^S202,T205^). The hippocampus was chosen due to the abundance of tau pathology in advanced AD [16]. pTau load in AD was confirmed to be significantly higher than controls (0.8 ± 0.2%, *p* = 0.0061) (Fig. 6b). SMOC1 colocalized with a subpopulation of pTau (9.6 ± 2.6%) in hippocampal AD tissue. Interestingly, although minimal pTau was observed in control cases (0.01 ± 0.006% load), SMOC1 colocalized with a higher proportion of pTau in controls than in AD (23.7 ± 6.1% compared to 9.6 ± 2.6%) (Fig. 6c). The extent of SMOC1 immunoreactivity in pTau aggregates correlated strongly with total pTau load (*r* = 0.955, *p* =  < 0.0001) (Fig. 6d). SMOC1 was observed to colocalize with all types of pTau morphologies present in AD tissue including neurofibrillary tangles, dystrophic neurites, neuropil threads, and neuritic plaques (Fig. 6e–g), with the majority of colocalization found within NFTs (77.3 ± 3.8%).

### SMOC1 interacts with Aβ and pTau in human AD brain

**Fig. 7:**
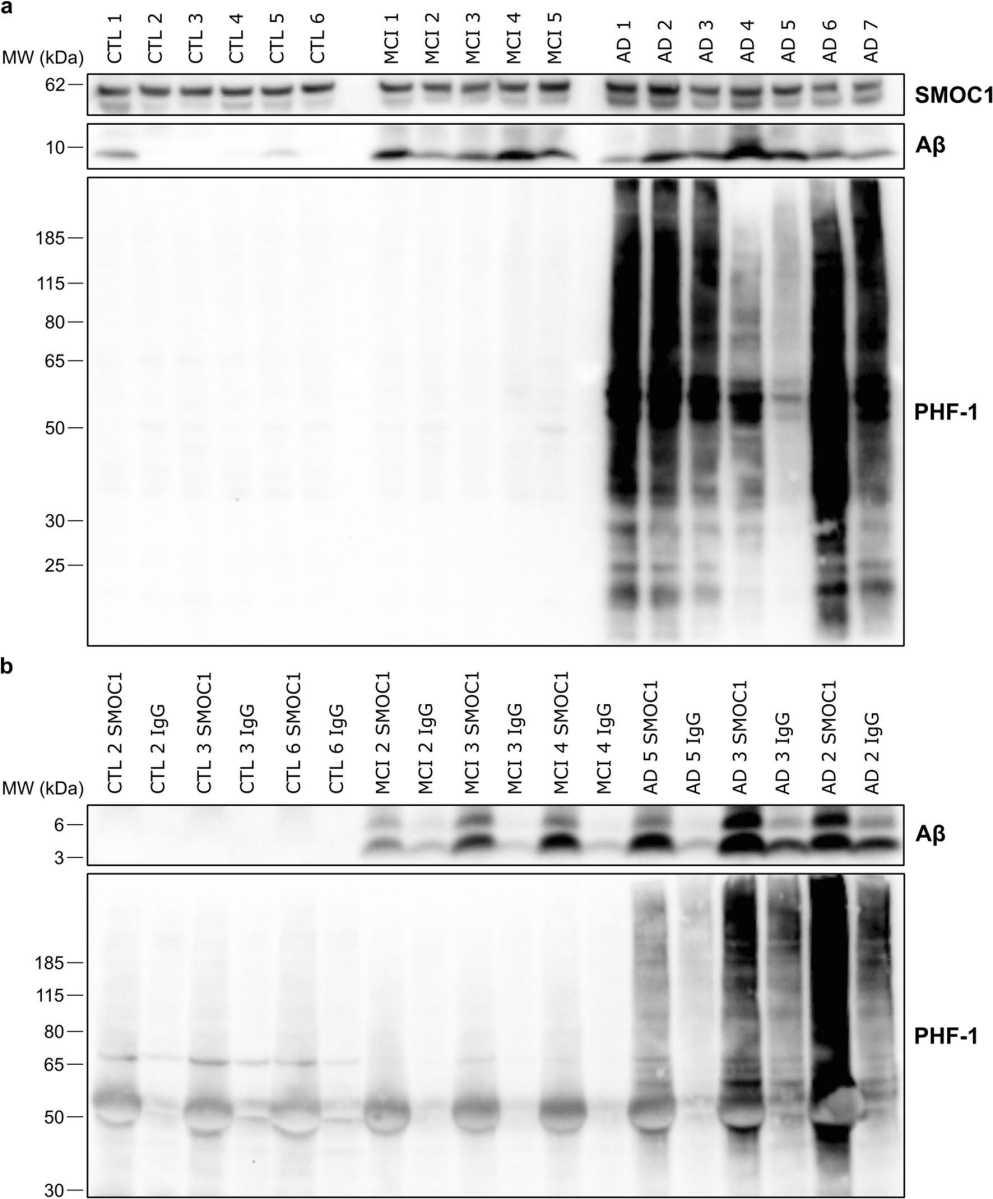
SMOC1 interacts with both Aβ and pTau in human brains. **a** Western blot results showing levels of SMOC1, Aβ, and PHF-1-immunoreactive pTau in human frontal cortex homogenate in *n* = 6 control, *n* = 5 MCI, and *n* = 7 AD cases. **b** Immunoprecipitation of SMOC1 and an IgG control antibody was performed in *n* = 3 cases per disease category that were selected to represent the diversity of pathology load in our cohort. Immunoprecipitation of SMOC1 pulled down both Aβ and PHF-1-immunoreactive pTau and the degree of SMOC1 interaction with each reflected the pathology load of each case

Given the strong colocalization of SMOC1 immunoreactivity observed with both Aβ and pTau pathology, we hypothesized that this colocalization resulted from a direct interaction between SMOC1 and Aβ or pTau in AD. To test this hypothesis, we used co-immunoprecipitation to determine if SMOC1 interacted with Aβ and PHF-1 immunoreactive pTau in human frontal cortex homogenate. Characterization of total SMOC1, Aβ, and PHF-1 was performed by immunoblotting *n* = 6 control, *n* = 5 MCI, and *n* = 7 AD cases (Fig. 7a). *n* = 3 control, MCI and AD cases were selected for immunoprecipitation to encompass the wide range of pathology load in our cohort. Immunoprecipitation of SMOC1 showed a strong interaction with both Aβ and PHF-1-immunoreactive pTau, that was not observed after pulldown with an IgG control antibody (Fig. 7b). Enrichment of both Aβ and PHF-1 immunoreactive pTau reflected total load in each case. Interestingly, SMOC1 appeared to interact with both monomers and dimers of Aβ, even in MCI cases when total Aβ load was comparatively low.

### SMOC1 inhibits Aβ_42_ fibril formation and alters fibril morphology

**Fig. 8:**
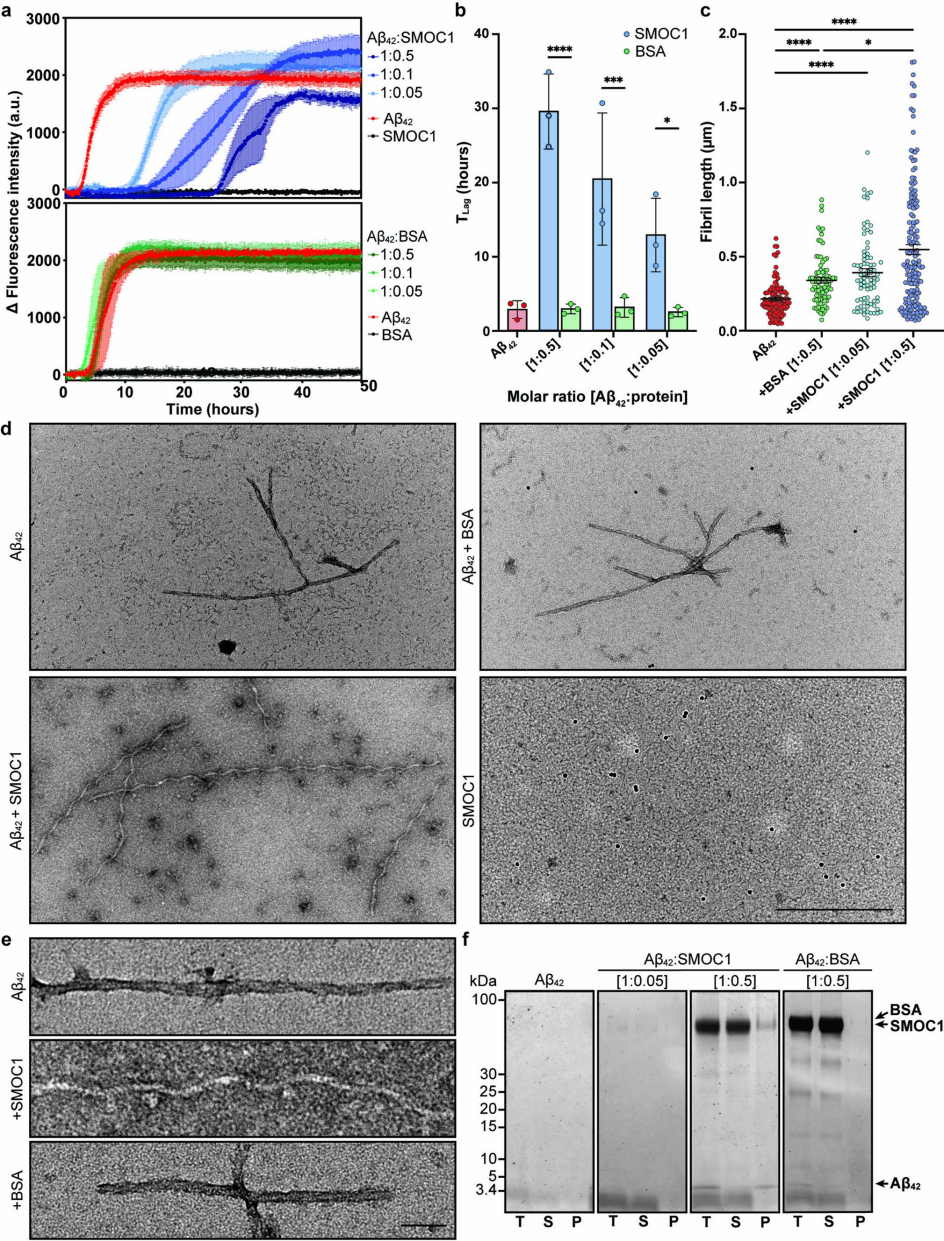
SMOC1 inhibits Aβ_42_ fibril aggregation and morphology in vitro. **a** The addition of SMOC1 delayed Aβ_42_ fibril formation in Thioflavin-T assays in a dose-dependent manner. Data show mean ± SD of *n* = 3 technical replicates. **b** SMOC1 significantly increased the lag time of Aβ_42_ fibril formation compared to BSA control at all tested concentrations. Data show mean ± SD of *n* = 3 technical replicates; * *p* < 0.05, *** *p* < 0.001, and **** *p* < 0.0001 determined by a two-way ANOVA with Tukey post hoc analysis. **c** Aβ_42_ fibril length was significantly increased in the presence of SMOC1 compared to BSA at the same concentration. * *p* < 0.05, **** *p* < 0.0001 determined by a Kruskal–Wallis test with uncorrected Dunn’s post hoc analysis. **d** Electron microscopy of Aβ_42_ fibrils show elongated fibrils and oligomeric structures when formed in the presence of SMOC1. Fibrils are negative for Ni–NTA NanoGold particles, suggesting that SMOC1 is not bound to Aβ_42_ fibrils at assay endpoint. Scale bar = 500 nm. **e** High magnification images show a clear corkscrew-like morphological change to Aβ_42_ fibrils in the presence of SMOC1. Scale bar = 50 nm. **f** SDS–PAGE analysis of assay samples shows that SMOC1 remains primarily in the soluble fraction at endpoint. T, total sample; S, soluble fraction; P, pellet

We next aimed to determine if SMOC1 influenced Aβ_42_ fibril formation in vitro. Thioflavin-T assays were performed in triplicate at 1:0.05, 1:0.1, and 1:0.5 molar ratios of monomeric Aβ_42_ to SMOC1 or BSA control. SMOC1 significantly delayed Aβ_42_ fibril formation at all concentrations tested in a dose–response manner. The lag phase to fibril assembly for Aβ_42_ alone under these conditions was 2.8 ± 1.2 h. Addition of SMOC1 resulted in a lag phase of 12.9 ± 5.0 h for an Aβ_42_:SMOC1 ratio of 1:0.05 (*p* = 0.019), 20.4 ± 9.0 h for a ratio of 1:0.1 (*p* = 0.0007), and 29.6 ± 5.1 h at a ratio of 1:0.5 (*p* < 0.0001) (Fig. 8a, b). We did not observe a similar lag effect after addition of the BSA control compared to Aβ_42_ alone (2.8 ± 1.2 h) at any concentration.

We were next interested to determine if SMOC1 altered Aβ_42_ fibril morphology. To assess this, ThT assay samples were collected at assay conclusion and probed with Ni–NTA-NanoGold particles to identify the presence of the His-tagged SMOC1 protein. TEM image analysis revealed that Aβ_42_ fibrils were significantly longer when formed in the presence of SMOC1 (0.55 ± 0.03 μm) compared to BSA (0.34 ± 0.02 μm, *p* = 0.02) at a 1:0.5 molar ratio (Fig. 8c). The presence of SMOC1 in a 10× lower molar ratio resulted in a slight lengthening of fibrils (0.39 ± 0.03 μm), indicating a dose-dependent effect of SMOC1 upon Aβ_42_ fibril length. Interestingly, fibrils formed in SMOC1 conditions exhibited a corkscrew-like morphology, which was distinct from the fibrils formed by pure Aβ_42_ or in the presence of BSA (Fig. 8d, e). Ni–NTA-NanoGold particles were not observed bound to the fibrils, suggesting that SMOC1 is not stably bound to Aβ_42_ fibrils at the assay endpoint. Spherical structures resembling Aβ_42_ oligomers were also observable in the SMOC1 + Aβ_42_ condition, suggesting that SMOC1 may delay Aβ fibril formation by interacting with on- or off-pathway oligomers. To confirm if SMOC1 was bound to Aβ_42_ fibrils, ThT assay samples were pelleted and the total, soluble and pellet fractions analyzed via SDS-PAGE. SMOC1 was observed primarily in the soluble fraction (Fig. 8f), confirming that the majority of SMOC1 was not bound to Aβ_42_ fibrils at assay endpoint. A similar result was observed when SMOC1 was added to mature, preformed Aβ_42_ fibrils (Supplementary Fig. 2), further supporting that SMOC1 does not bind stably to preformed Aβ_42_ fibrils.

## Discussion

We have shown that SMOC1 consistently colocalizes with a subpopulation of amyloid plaques in preclinical AD, MCI, and advanced AD. SMOC1 interacts with Aβ in AD human brain tissue, and delays Aβ aggregation in vitro. Additionally, SMOC1 interacts with phosphorylated tau and colocalizes with a subpopulation of tau pathology in human AD brain tissue. Together, our findings provide new evidence about the important role of SMOC1 in AD and provide new context for why SMOC1 is consistently reported as a leading fluid biomarker for early AD in proteomic studies [42, 48–50, 87, 109, 115, 117, 127].

The reason why SMOC1 is increased in AD is still unknown. One possibility is increased expression by OPCs in response to local Aβ pathology. SMOC1 is secreted by OPCs [46], which have been reported to become senescent around amyloid plaques in AD [126] and adopt a ‘reactive’ phenotype in an AD model [114] or in response to acute injury [2, 52, 82]. This could potentially result in altered expression profiles of OPC proteins including SMOC1; however, significant alterations in other OPC proteins in addition to SMOC1 would also be expected, which is not reflected in proteomic studies of human AD brain tissue [33–35, 65]. Instead, we hypothesize that the appearance of pathological Aβ species in the brain is the stimulus for increased SMOC1 expression. This is supported by our finding that SMOC1 accumulation in brain strongly correlated to plaque load, irrespective of disease stage, which is consistent with the highly significant correlation between SMOC1 and Aβ levels reported in brain tissue, CSF and plasma in AD [9, 36, 48–50, 63, 96, 98, 102, 115, 117, 127]. Increased SMOC1 expression is only reported in other neurodegenerative diseases when Aβ is present (e.g., Lewy Body Dementia) and correlates to Aβ pathology load rather than cognitive decline in AD [63, 64, 94, 117, 118, 127], suggesting that the SMOC1 increase is linked specifically to Aβ rather than AD pathogenesis more broadly. While further study is required to determine the mechanistic role of SMOC1 in AD, preliminary results from an independent group suggest that it may be neuroprotective as SMOC1 overexpression in a transgenic mouse model of AD significantly reduced amyloid plaque count without any accompanying neurotoxicity [3].

Interestingly, the increase of SMOC1 precisely coincides with the decrease of Aβ in the CSF in pre-symptomatic autosomal dominant AD, presumably reflecting Aβ sequestration into plaques in the brain [63]. These findings emphasize the strong association between SMOC1 and Aβ protein levels through AD progression; however, it is unclear why SMOC1 and Aβ protein levels change in opposing directions in the CSF. This is particularly notable given that SMOC1 increases proportionally to Aβ load in the AD brain [8, 9, 21, 53, 65–67]. Supporting these proteomic data, our co-immunoprecipitation results suggest a direct interaction between SMOC1 and Aβ in human brain tissue, and our immunostaining results clearly show that SMOC1 is sequestered in plaques in the brain. A possible explanation for the discrepancy between brain and CSF expression may be that only a fraction of the increased SMOC1 produced in AD binds to Aβ in plaques, while the remaining pool of soluble SMOC1 that does not complex with fibrillar Aβ is continuously cleared from the brain, thus accounting for the increased levels of SMOC1 in the CSF and plasma. Additional future studies are required to determine if this is the case.

We were interested to observe that SMOC1 only colocalized with a subpopulation of amyloid plaques in our study, consistent with previous observations [33, 98]. The frontal cortex showed significantly less colocalization of SMOC1 with Aβ plaques compared to the hippocampus and temporal cortex, perhaps reflective of lower levels of total OPCs within this region [84]. In all regions, the SMOC1-immunopositive plaque subpopulation was not defined by a particular morphology such as diffuse, cored, or neuritic plaques, and was not defined by layer-specific cortical location. In our previous study, we had hypothesized that this SMOC1-immunopositive plaque subpopulation could be defined by the presence of pyroglutamated Aβ [33]; however, our study here using double immunofluorescence showed that this was not the defining characteristic of this plaque subpopulation. Looking forward, it would be interesting to determine if this plaque population was defined by the presence of other types of Aβ (e.g. Aβ_40_, Aβ_42_, Aβ_38_, phosphorylated Aβ, etc.) or alternative factors such as localized association with OPCs.

The SMOC1 colocalization with tau pathology was surprising, as SMOC1 has not previously been detected in tau interactome analyses [35, 68, 111], and has not been reported in immunostaining studies examining SMOC1 in human AD brain tissue [9, 33, 98]. The interaction between SMOC1 and tau in AD has likely been overlooked in the previous studies due to the comparatively lower abundance of SMOC1 in tau pathology in comparison to plaques. Indeed, our results show only a low level (~10%) of SMOC1 colocalization with disease-associated phosphorylated tau observable in neurofibrillary tangles, dystrophic neurites, and neuritic plaques. Despite this relatively low colocalization, our co-immunoprecipitation results show a strong interaction with phosphorylated tau in human AD brain tissue. As we only examined the interaction between SMOC1 and PHF-1 immunoreactive tau in co-IP studies and AT8-immunoreactive tau in immunofluorescence studies, it is unclear if the observed interaction is specific to these types of tau, or if there is a broader interaction between SMOC1 and many subspecies of phosphorylated tau.

The calcium-binding actions of SMOC1 may indicate its role in AD. While the role of calcium-binding proteins in AD is not fully understood, calcium dyshomeostasis is an early feature of AD and other calcium-binding proteins have protective actions in AD [11, 13, 14, 23, 32, 37, 41, 43, 69–71, 73, 75, 78, 81].

Proteins with EF-hand calcium-binding domains similar to SMOC1 such as calbindin, parvalbumin, S100B, and calretinin have all been implicated in AD [7, 17, 20, 26, 51, 55–58, 78, 86, 93, 100, 103, 124]. Parallels between these proteins and SMOC1 may be used to inform on the actions of SMOC1 in AD. Of particular interest is S100B, which also inhibits Aβ_42_ fibril formation in vitro [26], binds Aβ in a calcium-dependent manner [103], and can rescue Aβ_42_-induced toxicity in SHSY5Y cells [26], supporting a neuroprotective role of calcium-binding proteins. The effect of calcium on the SMOC1-Aβ_42_ interaction was not investigated here, but would be interesting to explore in future studies examining the role of SMOC1 in AD.

To conclude, we have shown that SMOC1 interacts with both Aβ and phosphorylated tau in AD and is significantly associated with the three major neuropathological hallmarks of AD: plaques, tangles, and CAA. We hypothesize that SMOC1 is significantly increased in the brain in response to Aβ aggregation in the first stages of AD, which would provide context as to why SMOC1 has been consistently identified as an early AD biomarker. The ability of SMOC1 to inhibit Aβ aggregation suggests that SMOC1 could be a new potential therapeutic target for AD. However, future studies are essential to closely explore the role of SMOC1 in AD, particularly studies that examine if SMOC1 is neuroprotective in AD.

## Supplementary Information

Below is the link to the electronic supplementary material.

**Supplementary Figure 1: SMOC1 distribution in plaques is not determined by plaque size. **SMOC1 immunofluorescence in plaques did not correlate with amyloid plaque size. Nonparametric Spearman correlation of *n* = 10 preclinical AD, *n* = 12 MCI and *n* = 12 temporal cortex sections. Dotted lines represent 95% confidence intervals. (TIFF 3576 KB)

**Supplementary Figure 2: SMOC1 does not bind to mature Aβ42 fibrils**. (**a**) SMOC1 remains in the soluble fraction after incubation with mature Aβ42 fibrils. (**b**) Electron microscopy of Aβ42 fibrils show no binding of NanoGold to fibrils, and no change of fibril morphology with SMOC1 addition. Scale bar = 500 nm. (TIF 4819 KB)

**Supplementary Figure 3: SMOC1 antibody validation.** (**a**) Three SMOC1 antibodies were tested for Western Blot application on SMOC1-tGFP overexpressing SHSY5Y lysates. ab200219 (Abcam) was determined to give the clearest signal compared to PA5-31392 (ThermoFisher) or PA5-113408 (ThermoFisher). All antibodies were tested at 1:500 in 2.5% skim milk. (**b**) ab200219 (Abcam) was tested with blocking buffers of 2.5% skim milk, 2.5% skim milk + 2.5% NGS, and 2.5% BSA on human brain homogenate (1:500). The combination buffer of skim milk + NGS was determined to give the least non-specific signal. (**c**) To confirm antibody specificity, oligodendrocyte MO3.13 and neuroblastoma SHSY5Y cells were transfected with HA-tagged SMOC1 plasmid, incubated for 48 hours and lysates collected. SMOC1 detection was then examined in lysates, human brain homogenate, and SMOC1 recombinant peptides (shorter exposure). ab20219 detected a strong 62 kDa band in the SMOC1 peptide and SMOC1-overexpression conditions, confirming antibody specificity. (**d**–**f**) Three SMOC1 antibodies were tested for immunofluorescence in human AD FFPE brain tissue. All antibodies were tested at 1:100 on the same case. ab200219 (Abcam) was determined to give the best immunofluorescent signal in plaques (arrows) (**d**) compared to PA5-31392 (ThermoFisher) (**e**) or WH0064093M3 (Sigma-Aldrich) (**f**). Scale bars = 200 μm (overviews), 20 μm (inserts). NGS; normal goat serum, BSA; bovine serum albumin. (PNG 5901 KB)

**Supplementary Figure 4: High magnification visualisation of SMOC1 colocalization with Aβ.** Plaques were imaged on a Nikon C2 at 100× magnification over 6 μm depth (0.1 μm steps). Images were deconvoluted using Huygens Professional and visualized in ImageJ. SMOC1 immunofluorescence (green) was observed to closely colocalize with amyloid fibrils (red) within plaques. Scale bars = 20 μm. (TIFF 6392 KB)

## Data Availability

Data is provided within the manuscript or supplementary information files.
