# Supplementary figures and images for: SMOC1 colocalizes with Alzheimer’s disease neuropathology and delays Aβ aggregation
Source: Acta Neuropathol. 2024 Nov 25;148(1):72. doi: 10.1007/s00401-024-02819-6 (PMC11588930; doi:10.1007/s00401-024-02819-6)

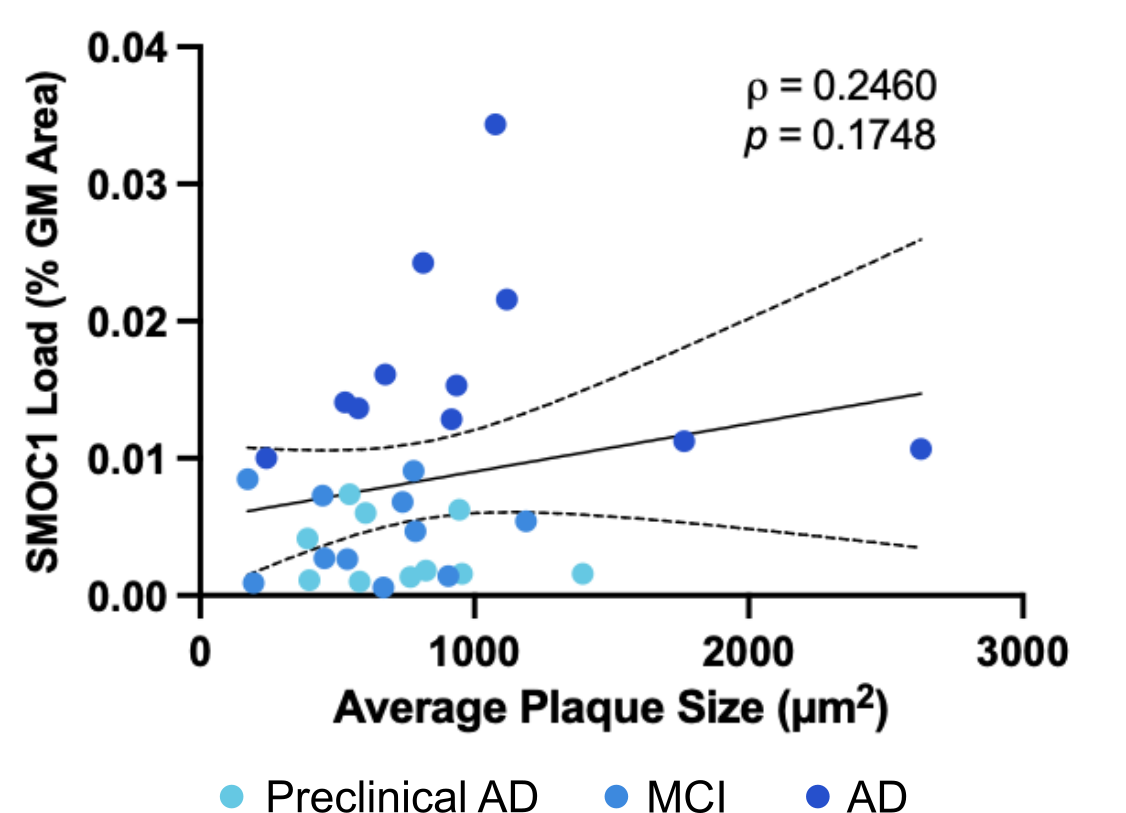

Supplement: Supplementary file 1 — Supplementary Figure 1: SMOC1 distribution in plaques is not determined by plaque size. SMOC1 immunofluorescence in plaques did not correlate with amyloid plaque size. Nonparametric Spearman correlation of n = 10 preclinical AD, n = 12 MCI and n = 12 temporal cortex sections. Dotted lines represent 95% confidence intervals. (TIFF 3576 KB) [file 401_2024_2819_MOESM1_ESM.tiff]

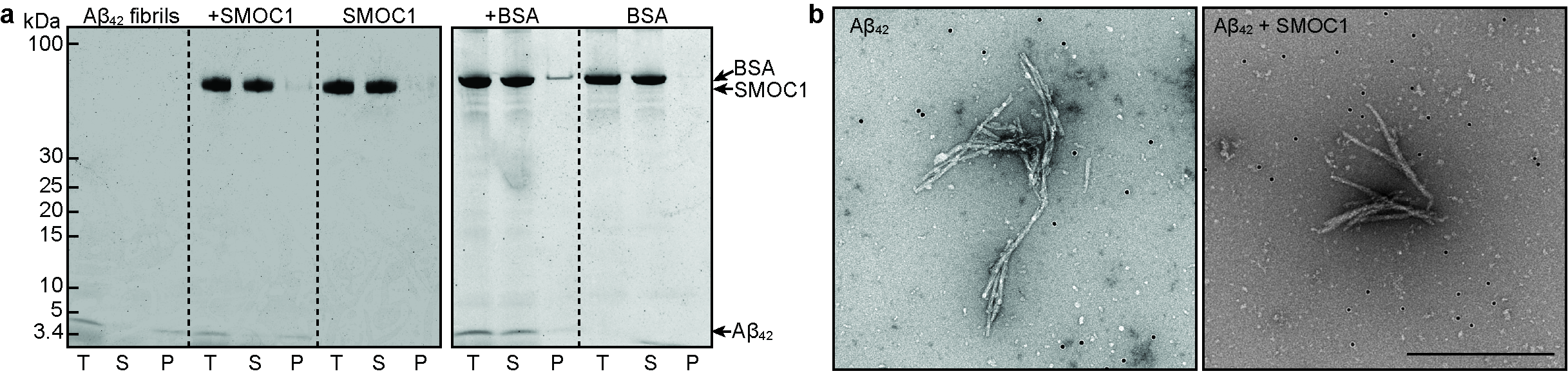

Supplement: Supplementary file 2 — Supplementary Figure 2: SMOC1 does not bind to mature Aβ42 fibrils. (a) SMOC1 remains in the soluble fraction after incubation with mature Aβ42 fibrils. (b) Electron microscopy of Aβ42 fibrils show no binding of NanoGold to fibrils, and no change of fibril morphology with SMOC1 addition. Scale bar = 500 nm. (TIF 4819 KB) [file 401_2024_2819_MOESM2_ESM.tif]

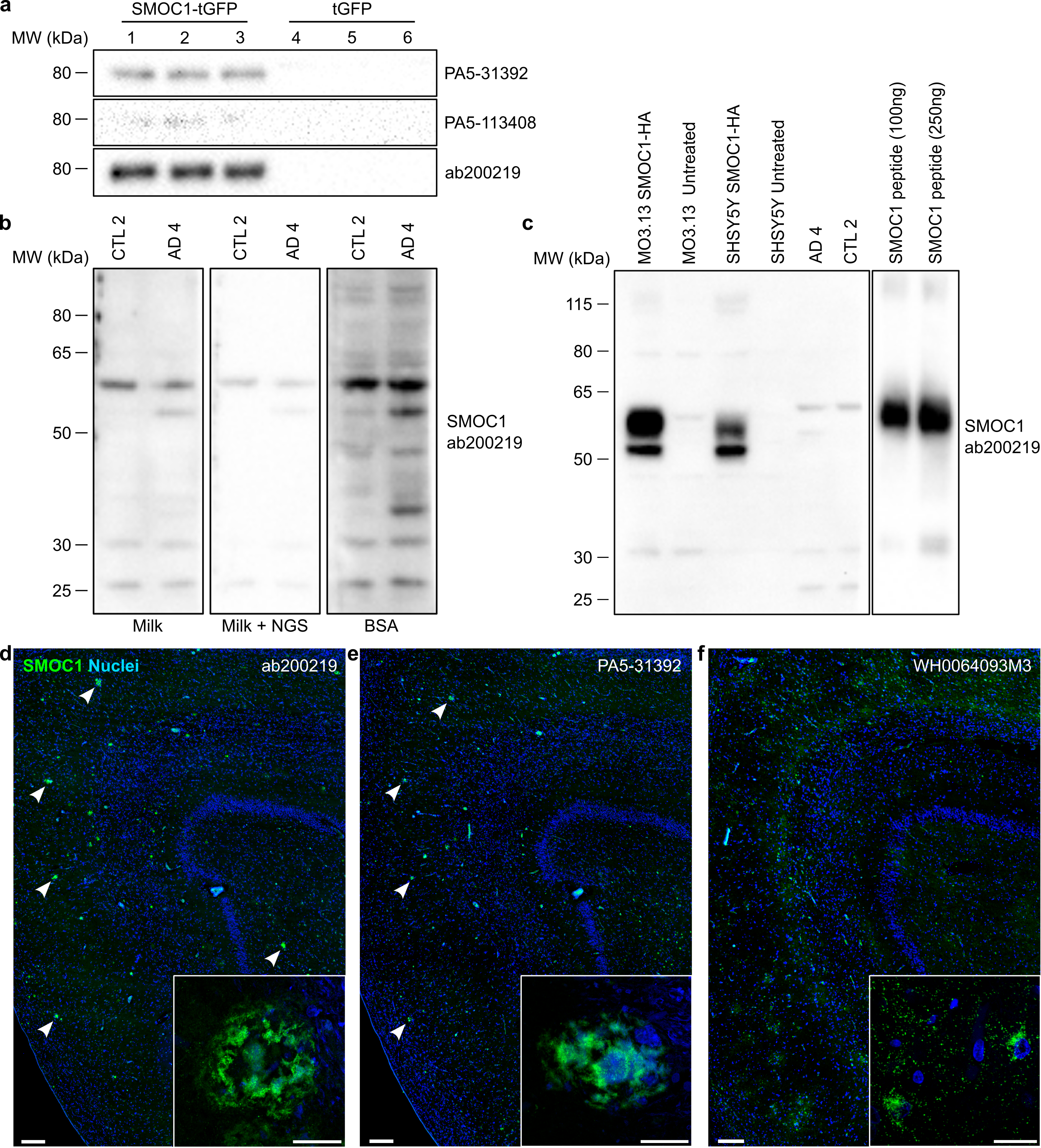

Supplement: Supplementary file 3 — Supplementary Figure 3: SMOC1 antibody validation. (a) Three SMOC1 antibodies were tested for Western Blot application on SMOC1-tGFP overexpressing SHSY5Y lysates. ab200219 (Abcam) was determined to give the clearest signal compared to PA5-31392 (ThermoFisher) or PA5-113408 (ThermoFisher). All antibodies were tested at 1:500 in 2.5% skim milk. (b) ab200219 (Abcam) was tested with blocking buffers of 2.5% skim milk, 2.5% skim milk + 2.5% NGS, and 2.5% BSA on human brain homogenate (1:500). The combination buffer of skim milk + NGS was determined to give the least non-specific signal. (c) To confirm antibody specificity, oligodendrocyte MO3.13 and neuroblastoma SHSY5Y cells were transfected with HA-tagged SMOC1 plasmid, incubated for 48 hours and lysates collected. SMOC1 detection was then examined in lysates, human brain homogenate, and SMOC1 recombinant peptides (shorter exposure). ab20219 detected a strong 62 kDa band in the SMOC1 peptide and SMOC1-overexpression conditions, confirming antibody specificity. (d–f) Three SMOC1 antibodies were tested for immunofluorescence in human AD FFPE brain tissue. All antibodies were tested at 1:100 on the same case. ab200219 (Abcam) was determined to give the best immunofluorescent signal in plaques (arrows) (d) compared to PA5-31392 (ThermoFisher) (e) or WH0064093M3 (Sigma-Aldrich) (f). Scale bars = 200 μm (overviews), 20 μm (inserts). NGS; normal goat serum, BSA; bovine serum albumin. (PNG 5901 KB) [file 401_2024_2819_MOESM3_ESM.png]

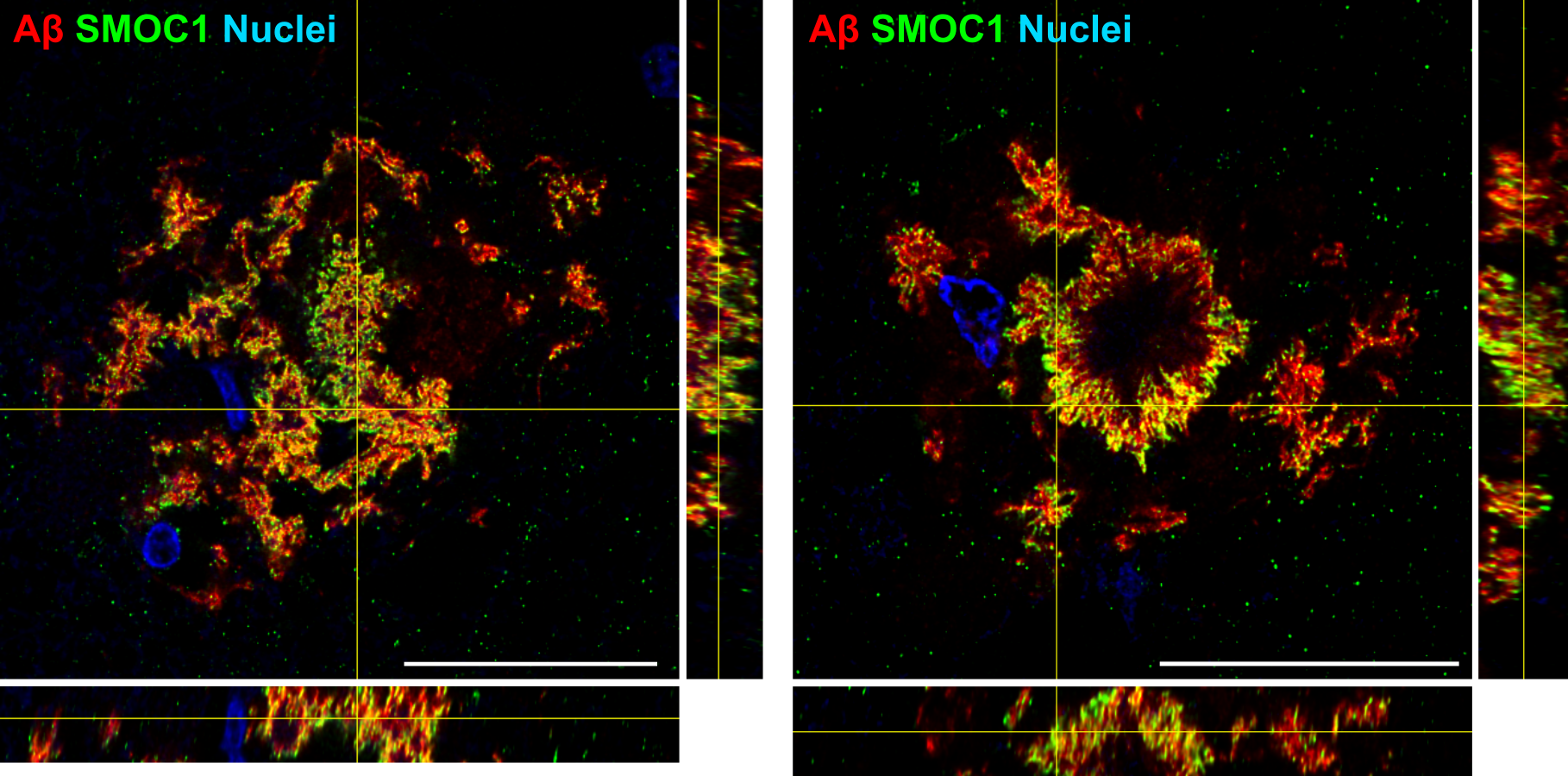

Supplement: Supplementary file 4 — Supplementary Figure 4: High magnification visualisation of SMOC1 colocalization with Aβ. Plaques were imaged on a Nikon C2 at 100× magnification over 6 μm depth (0.1 μm steps). Images were deconvoluted using Huygens Professional and visualized in ImageJ. SMOC1 immunofluorescence (green) was observed to closely colocalize with amyloid fibrils (red) within plaques. Scale bars = 20 μm. (TIFF 6392 KB) [file 401_2024_2819_MOESM4_ESM.tiff]
